# The role of *neuromedin U* in adiposity regulation. Haplotype analysis in European children from the IDEFICS Cohort

**DOI:** 10.1371/journal.pone.0172698

**Published:** 2017-02-24

**Authors:** Francesco Gianfagna, Claudio Grippi, Wolfgang Ahrens, Mark E. S. Bailey, Claudia Börnhorst, Stefan De Henauw, Ronja Foraita, Anna C. Koni, Vittorio Krogh, Staffan Mårild, Dénes Molnár, Luis Moreno, Yannis Pitsiladis, Paola Russo, Alfonso Siani, Michael Tornaritis, Toomas Veidebaum, Licia Iacoviello

**Affiliations:** 1 Laboratory of Molecular and Nutritional Epidemiology, Department of Epidemiology and Prevention, IRCCS Istituto Neurologico Mediterraneo Neuromed, Pozzilli, Isernia, Italy; 2 EPIMED Research Center, Department of Clinical and Experimental Medicine, University of Insubria, Varese, Italy; 3 Leibniz Institute for Prevention Research and Epidemiology – BIPS, Bremen, Germany; 4 Faculty of Mathematics and Computer Science, Institute of Statistics, Bremen University, Bremen, Germany; 5 School of Life Sciences, College of Medical, Veterinary and Life Sciences, University of Glasgow, Glasgow, United Kingdom; 6 Department of Public Health, Faculty of Medicine and Health Sciences, Ghent University, Ghent, Belgium; 7 Department of Preventive and Predictive Medicine, Fondazione IRCCS Istituto Nazionale dei Tumori, Milan, Italy; 8 Dept. of Paediatrics, Inst. of Clinical Sciences, Sahlgrenska Academy at Gothenburg University, Gothenburg, Sweden; 9 Department of Paediatrics, Medical Faculty, University of Pécs, Pécs, Hungary; 10 GENUD (Growth, Exercise, Nutrition and Development) Research Group, University of Zaragoza, Zaragoza, Spain; 11 Centre for Sport and Exercise Science and Medicine, University of Brighton, Brighton, United Kingdom; 12 Unit of Epidemiology & Population Genetics, Institute of Food Sciences, CNR, Avellino, Italy; 13 Research & Education Institute of Child Health, Strovolos, Cyprus; 14 Department of Chronic Diseases, National Institute for Health Development, Tallinn, Estonia; McMaster University, CANADA

## Abstract

**Background and aims:**

Neuromedin U (NMU) is a hypothalamic neuropeptide with important roles in several metabolic processes, recently suggested as potential therapeutic target for obesity. We analysed the associations between *NMU* gene variants and haplotypes and body mass index (BMI) in a large sample of European children.

**Methods and results:**

From a large European multi-center study on childhood obesity, 4,528 children (2.0–9.9 years, mean age 6.0±1.8 SD; boys 52.2%) were randomly selected, stratifying by age, sex and country, and genotyped for tag single nucleotide polymorphisms (SNPs; rs6827359, T:C; rs12500837, T:C; rs9999653,C:T) of *NMU* gene, then haplotypes were inferred. Regression models were applied to estimate the associations between SNPs or haplotypes and BMI as well as other anthropometric measures. BMI was associated with all *NMU* SNPs (*p*<0.05). Among five haplotypes inferred, the haplotype carrying the minor alleles (CCT, frequency = 22.3%) was the only associated with lower BMI values (beta = -0.16, 95%CI:-0.28,-0.04, p = 0.006; *z*-score, beta = -0.08, 95%CI:-0.14,-0.01, p = 0.019) and decreased risk of overweight/obesity (OR = 0.81, 95%CI:0.68,0.97, p = 0.020) when compared to the most prevalent haplotype (codominant model). Similar significant associations were also observed using the same variables collected after two years’ time (BMI, beta = -0.25, 95%CI:-0.41,-0.08, p = 0.004; *z*-score, beta = -0.10, 95%CI:-0.18,-0.03, p = 0.009; overweight/obesity OR = 0.81, 95%CI:0.66,0.99, p = 0.036). The association was age-dependent in girls (interaction between CCT haplotypes and age, *p* = 0.008), more evident between 7 and 9 years of age. The CCT haplotype was consistently associated with lower levels of fat mass, skinfold thickness, hip and arm circumferences both at T0 and at T1, after adjustment for multiple testing (FDR-adjusted *p*<0.05).

**Conclusions:**

This study shows an association between a *NMU* haplotype and anthropometric indices, mainly linked to fat mass, which appears to be age- and sex-specific in children. Genetic variations within or in linkage with this haplotype should be investigated to identify functional variants responsible for the observed phenotypic variation.

## Introduction

Neuromedin U (NMU) is a neuropeptide mainly expressed in brain, adipose tissue and gastroenteric tract. NMU plays important roles in appetite regulation, energy homeostasis, gastric secretion, smooth muscle contraction and bone remodeling, as well as in progression of different types of cancer. This neuropeptide might therefore be a novel target for the treatment of many diseases, and some analogs have already been developed for treatment of obesity and metabolic disturbances [1].

Some evidence suggests a strong impact of NMU on the regulation of eating behavior and adiposity. Transgenic mice models showed that *Nmu*-null mice are hyperphagic and have a decreased energy expenditure, resulting in increased adiposity, decreased insulin sensitivity and increased bone mass [2,3]. Experimental studies showed that intracerebroventricular administration of NMU decreases food intake and feeding-associated behavior in rats, leading to food intake reduction and loss of body weight, as well as increased locomotor activity and increased heat production [4–7]. At the same time, peripheral administration of NMU lead to reduced NMU concentrations in the hypothalamus [8]. All these findings suggest that NMU acts through both central and peripheral mechanisms. However, the specific mechanisms of action still remain to be elucidated.

Although many studies using animal models are available, there are few studies on the role of NMU in humans. A genome-wide linkage study in Europeans found suggestive genetic linkage of obesity to the region containing *NMU* on chromosome 4 [9]. Furthermore, in a candidate gene study, Hainerovà et al. [10] showed an association between a missense polymorphism of *NMU* gene and adiposity parameters in a Danish adult population and suggested a linkage with childhood obesity. No genome-wide association studies (GWAs) on obesity identified signals for single nucleotide polymorphisms (SNPs) near the *NMU* region. However, GWAs do not have sufficient resolution to capture all genetic variations and, although the heritability of body mass index (BMI) is 40–70%, the SNPs identified associated in GWAs explained only 2.7% of the total variance [11]. An alternative way to study the association between genetic variations and phenotypes is haplotype analysis. This approach allows to capture most of the existing combinations of genetic variants found in a population (haplotypes), confined to specific regions delimited by historical recombination events (haplotype blocks). Functional genetic variants in haplotypes of small haplotype blocks, which have a low probability to be captured by spread markers used in GWAs, could reasonably be responsible for the ‘missing heritability’ [12]. The DNA sequence of *NMU* and its haplotype blocks are short, as well as highly conserved across different species. For this reason, a high impact on peptide function is expected for human genetic variations. Following the haplotype approach, we have recently identified an association between variations in a *NMU* haplotype block and bone stiffness in a children population [13].

In the present study we investigated the associations between variations in this *NMU* haplotype block and BMI in a large sample of European children of the IDEFICS (Identification and prevention of Dietary- and lifestyle-induced health EFfects In Children and infantS) study [14]. Because the *NMU* gene expression changes from infancy to puberty [15], we investigated whether also the potential associations change during child growth due to interactions between genetic and age-specific factors. Furthermore, to identify the mechanisms through which *NMU* regulates adiposity, we also considered the potential association with several fat and fat-free mass related indices in addition to BMI.

## Material and methods

### Study population

IDEFICS is a large European multi-center study on childhood obesity [14]. A cohort of 16,224 children aged 2.0–9.9 years was recruited in a population-based survey between September 2007 and May 2008 (T0), in eight European countries (Belgium, Cyprus, Estonia, Germany, Hungary, Italy, Spain and Sweden). A community oriented intervention program for primary prevention of obesity was implemented, using an integrated set of actions at different levels of society to facilitate the adoption of a healthy lifestyle [16]. Children were allocated to either control or intervention group and were followed up for two years (T1, 2009–2010). Ethical approval was obtained by the ethical committees belonging to each of the eight centers engaged in the fieldwork: Ethics Committee, University Hospital, Gent, Belgium; Cyprus National Bioethics Committee, Strovolos, Cyprus; Tallinn Medical Research Ethics Committee, Tallinn, Estonia; Ethics Committee, University of Bremen, Bremen, Germany; Egészségügyi Tudományos Tanács, Pécs, Hungary; Comitato Etico, ASL Avellino, Avellino, Italy; Comité Ético de Investigación, Clínica de Aragón (CEICA), Zaragoza, Spain; Regional Ethics Committee, University of Gothenburg, Gothenburg, Sweden. Both children and their parents gave oral (children) and written (parents) informed consent.

For this analysis, a subgroup of 4,678 samples was randomly selected from the total study population of European descent children, stratifying by age, sex and country (about 600 subjects from each country) [13,17,18].

### Data collection

Data were collected at baseline (T0) and after two years (T1). Height and weight were measured using a standard clinical Seca 225 stadiometer (Seca, Hamburg, Germany) to the nearest 0.1 cm and a scale (BC 420 SMA; Tanita, Amsterdam, The Netherlands) to the nearest 0.1 kg, respectively. Children wore underwear clothes, without shoes. The Tanita scale also measured leg-to-leg impedance. BMI was calculated as weight(kg)/height(m)^2^. For the definition of overweight/obesity, children were grouped into categories using the BMI cut-points defined by Cole et al. [19]. Fat-free mass (FFM) was calculated using the Tyrrell et al. [20] formula: FFM(kg) = 0.31×height(cm)^2^/impedance(cm²/Ω)+0.17×height(cm)+0.11×weight(kg)+0.942×S-14.96, where S = 1 for girls and 2 for boys. Waist and hip circumference was measured with an inelastic tape (Seca 200), precision 0.1 cm, range 0–150 cm, with the subject in a standing position. Tricipital and subscapular skinfold thickness was measured with a Holtain caliper (Holtain, Holtain Ltd, Pembrokeshire, UK, range 0±40 mm). Measures were taken twice on the right hand side of the body and the mean was used in subsequent analyses. All measurements were collected by standardized protocols across centers and intra- and inter-observer reliability was carefully checked [21].

### Genotyping

DNA was extracted from saliva samples (Oragene DNA Self-Collection Kit, OG-300/OG-250; DNA Genotek Inc., Kanata, Ontario, Canada) [22]. Three tag SNPs (rs6827359, rs12500837, rs9999653; intronic regions) of the central haplotype block among the three main blocks of the *NMU* gene (chr4, 55595229–55636698, GRCh38.p7 assembly; S1 Fig) were selected from the Caucasian HapMap Project data using the Tagger Pairwise method of Haploview software (version 4.1; Broad Institute, Cambridge, MA, USA) [23]. Tag SNP selection criteria were: (i) a pairwise r^2^ threshold of 0.8 (S1 Table); (ii) SNPs defining haplotypes with frequency >10%. The selected tag SNPs cover only 33% of genetic variance of the whole haplotype block, however they discriminate all the four main haplotypes found in Caucasian population (S1 Fig panel c). The analysis based on the selected haplotype frequency and sample size is able (80% power, α = 0.05) to detect a true difference of ±0.1 in BMI (as expected from previous association studies) with a glm regression using posterior probabilities of pairs of haplotypes in a codominant model. The SNPs were genotyped by a multiplexed end-point assay. The allelic discrimination was performed by 7500 Fast Real-Time System (Applied Biosystems). The genotyping success rate was on average 97.6% and a randomly selected sample (5%) was newly genotyped for all SNPs, with 100% concordance.

### Statistical analysis

Hardy-Weinberg equilibrium (HWE) was assessed with the chi-square test. Distribution of continuous variables was assessed using the Kolmogorov–Smirnov test and *z*-scores or log-transformed variables were used where appropriate (BMI, weight, skinfolds and waist, hip and arm circumferences). The best genetic model was checked for each genotype–phenotype association, testing dominance deviation from additivity and considering the additive model as default [18].

Ordinary least square regression was applied to estimate the associations between BMI or BMI z-scores and gene variants with SAS software (v9.3,SAS Institute Inc., Cary, NC). The Haplo.stats package of R software (v3.2.1; https://www.R-project.org/), was used to estimate the haplotype frequencies and to verify the associations between haplotype and phenotype (haplo.glm function, the most prevalent haplotype as reference). Haplotypes with frequencies lower than 1% were excluded. Logistic regression was used to estimate the association between gene variants and BMI categories. The analyses were performed using data collected at T0, then the analysis was repeated for data newly collected at T1, to cross-sectionally replicate the haplotype-phenotype associations observed at T0. To evaluate if the associations were dependent on age, the interaction between haplotypes and age was also tested, adding their interaction term (haplotype*age as continuous) to the full regression models. The BMI values and *z*-scores were plotted in a graph to show changes over time, through a locally weighted regression (PROC SGPLOT with LOESS statement in SAS) using a scatterplot smoothing method that automatically determines the optimal smoothing parameter [24]. Age, sex, country and (for T1) intervention (yes/no) were considered as covariates.

Regression analyses were performed to evaluate associations between genotypes/haplotypes and all anthropometric indices. Since several secondary associations were tested, the Benjamini-Hochberg false discovery rate (FDR) [25] was used to adjust the results for multiple comparisons, using PROC MULTTEST in SAS. A FDR-adjusted *p* value (*p*FDR) <0.05 was considered as statistically significant.

## Results

### Population characteristics

Characteristics of the study population are listed in Table 1. Children with at least one SNP successfully genotyped in the NMU gene were 4,528 (T0 sample). Among them, 3,277 (72.4%; intervention group: N = 1598, 48.8% of the T1 sample) returned for follow-up at T1. At T0 (mean age 6.0±1.8 SD, boys 52.2%), the mean BMI was 16.35(±2.33 SD; BMI *z*-score 0.18±1.29 SD), while at T1 (mean age 8.0±1.8 SD, boys 52.0%) was 17.06 (±2.95; BMI *z*-score 0.34±1.31 SD). All genotypes were in HWE and the minor allele frequencies (MAF) were similar to values reported in the HapMap database for Caucasians (Table 2). Five haplotypes were inferred (Table 3; wild-type haplotype 43.9%).

**Table 1:** Anthropometric characteristics of N = 4,528 children with genotype data recruited at baseline of the IDEFICS study.

	T0				T1			
	T0	All	F (47.8%)	M (52.2%)	T1	All	F (48.0%)	M (52.0%)
	**N**	**Mean±SD**	**Mean±SD**	**Mean±SD**	**N**	**Mean±SD**	**Mean±SD**	**Mean±SD**
Age (years)	4,528	6.02±1.80	6.04±1.79	6.01±1.81	3,277	8.03±1.78	8.06±1.77	8.01±1.79
Body Mass Index (Kg/m^2^)	4,528	16.35±2.33	16.34±2.35	16.36±2.31	3,277	17.05±2.95	17.05±2.91	17.07±2.98
BMI *z*-score	4,528	0.18±1.29	0.17±1.32	0.20±1.27	3,277	0.34±1.31	0.33±1.32	0.34±1.30
Weight (kg)	4,528	22.96±6.89	22.73±6.88	23.18±6.88	3,277	29.40±9.02	29.18±9.00	29.61±9.04
Fat mass (kg)	4,463	7.21±3.23	7.86±3.24	6.63±3.11	3,227	9.10±4.98	9.83±4.88	8.42±4.98
Fat free mass (kg)	4,454	15.77±4.70	14.87±4.57	16.62±4.71	3,227	20.27±5.08	19.31±4.91	21.17±5.08
Triceps+subscapular skinfolds (cm)	4,391	18.23±7.33	19.56±7.63	17.02±6.81	3,180	20.66±9.75	22.19±9.76	19.24±9.52
Triceps skinfold (mm)	4,416	11.16±3.94	11.98±4.04	10.41±3.68	3,184	12.41±5.03	13.30±4.97	11.60±4.95
Subscapular skinfold (mm)	4,413	7.09±3.81	7.60±4.08	6.61±3.49	3,183	8.25±5.16	8.91±5.29	7.65±4.96
Waist circumference (cm)	4,527	54.24±6.77	53.92±6.76	54.52±6.76	3,222	58.45±8.15	58.04±8.09	58.85±8.20
Hip circumference (cm)	4,527	62.77±7.90	63.02±7.90	62.54±7.90	3,205	68.67±8.84	69.05±8.85	68.32±8.83
Waist/hip ratio	4,526	0.87±0.05	0.86±0.06	0.87±0.05	3,200	0.85±0.05	0.84±0.05	0.86±0.05
Mid-upper arm circumference (cm)	4,497	18.77±2.45	18.87±2.46	18.68±2.43	3,016	20.26±3.10	20.36±3.09	20.16±3.10
**BMI categories**	**N**	**%**	**N(%)**	**N(%)**	**N**	**%**	**N(%)**	**N(%)**
*Thinness*	478	10.6%	208(9.6%)	270(11.4%)	324	9.9%	151(9.6%)	173(10.2%)
*Normal weight*	3,234	71.4	1,527(70.6%)	1,707(72.2%)	2,256	68.8%	1,081(68.7%)	1,175(69.0%)
*Overweight*	541	11.9%	292(13.5%)	249(10.5%)	496	15.2%	242(15.4%)	254(14.9%)
*Obese*	275	6.1%	137(6.3%)	138(5.8%)	201	6.1%	99(6.3%)	102(6.0%)

**Table 2:** Allele frequencies and Hardy-Weinberg equilibrium of the *NMU* SNPs (n = 4,528 with at least one SNP successfully genotyped; T0).

SNP	N	Major:minor allele	Homozygous (major allele)	Heterozygous _	Homozygous (minor allele)	HWE (*p*)	MAF (%)	CEU (%)
rs6827359	4508	T*:C	1211 (26.9%)	2199 (48.8%)	1098 (24.3%)	0.11	49	40
rs12500837	4505	T*:C	2580 (57.3%)	1656 (36.7%)	269 (6.0%)	0.91	24	21
rs9999653	4506	C:T*	967 (21.5%)	2212 (49.1%)	1327 (29.4%)	0.42	54	49

HWE: Hardy-Weinberg equilibrium, p value; MAF: Minor Allele Frequency in the sample; CEU: MAF in CEPH population (Utah residents with Northern and Western European Ancestry) from International HapMap Project.

* Ancestral allele

**Table 3:** Haplotype frequencies in the whole sample and stratified for BMI categories (n = 4,528 with at least one SNP successfully genotyped; T0).

Alleles			Haplotype frequencies				
rs6827359	rs12500837	rs9999653	Whole sample	Thinness	Normal weight	Overweight	Obese
T	T	C	43.9%	44.6%	43.3%	46.8%	46.0%
C	T	T	24.4%	22.9%	24.4%	24.5%	24.0%
C	C	T	22.3%	23.3%	22.7%	20.0%	22.2%
T	T	T	7.2%	7.3%	7.4%	7.1%	5.7%
C	C	C	2.0%	1.8%	2.2%	1.5%	2.2%

Rare haplotypes with frequency lower than 1% were not considered (CTC, TCC, TCT, accounting for less than 0.2%)

### Association with BMI

The additive model was the best genetic model for all genotype- and haplotype-phenotype associations. BMI was associated with all *NMU* SNPs (Table 4). Among haplotypes, CCT (haplotype frequency 22.3%; heterozygotes 34.8%, homozygotes 5.0%) showed significant negative associations with BMI (β = -0.16 for each haplotype copy, 95%CI:-0.28,-0.04, *p* = 0.006; *z*-scores -0.08, 95%CI:-0.14,-0.01, *p* = 0.019), as compared to the wild type (TTC, 43.9%), while the other haplotypes did not (S2 Table). Analysis at T1 showed similar results (-0.25; 95%CI:-0.41,-0.08, *p* = 0.004; z-scores -0.10, 95%CI:-0.18,-0.03, *p* = 0.009; Table 4). Logistic regression analyses showed significant decreased risk of being overweight or obese in the presence of variant alleles in two of the three SNPs and the CCT haplotype (OR = 0.81; 95%CI:0.68,0.97; p = 0.020), confirmed at T1 (Table 4). The analyses were repeated using the haplotype carrying the ancestral alleles as reference (TTT, haplotype frequency 7.2%), obtaining similar results (S2 Table). Results of association analyses using dominant and recessive models are also reported in S2 Table.

**Table 4:** Associations between *NMU* genotypes and haplotypes and BMI, other anthropometric parameters and BMI categories.

	rs6827359 C^	rs12500837 C^	rs9999653 T^	CCT haplotype		CCT haplotype (T1)	
**Main variable**	**Beta (95%CI)**	**Beta (95%CI)**	**Beta (95%CI)**	**Beta (95%CI)**	***p***	**Beta (95%CI)**	***p***
Body Mass Index^§^	-0.12 (-0.21,-0.03)*	-0.13 (-0.24,-0.03)*	-0.10 (0.005,0.19)*	-0.16 (-0.28,-0.04)	**0.006**	-0.25 (-0.41,-0.08)	**0.004**
BMI *z*-scores	-0.07 (-0.12,-0.02)*	-0.06 (-0.12,-0.002)*	-0.05 (0.002,0.10)*	-0.08 (-0.14,-0.01)	**0.019**	-0.10 (-0.18,-0.03)	**0.009**
**Other variables**	**Beta (95%CI)**	**Beta (95%CI)**	**Beta (95%CI)**	**Beta (95%CI)**	***p*FDR**	**Beta (95%CI)**	***p*FDR**
Weight^§^ (kg)	-0.26 (-0.44,-0.08)*	-0.27 (-0.48,-0.05)*	-0.18 (0.002,0.37)	-0.31 (-0.55,-0.08)	**0.028**	-0.59 (-0.99,-0.19)	**0.020**
*Fat mass (kg)*	-0.17 (-0.29,-0.06)*	-0.15 (-0.29,-0.01)*	-0.14 (0.02,0.26)*	-0.20 (-0.36,-0.05)	**0.022**	-0.46 (-0.73,-0.19)	**0.010**
*Fat free mass (kg)*	-0.08 (-0.17, 0.01)	-0.10 (-0.20,0.01)	-0.03 (-0.06,0.12)	-0.10 (-0.23,0.04)	0.16	-0.19 (-0.41,-0.02)	0.07
Skinfolds^§^ (tricipital *plus* subscapular, mm)	-0.33 (-0.62,-0.05)*	-0.48 (-0.81,-0.14)*	-0.30 (0.01,0.59)*	-0.55 (-0.93,-0.18)	**0.013**	-0.72 (-1.29,-0.16)	**0.022**
*Tricipital*^§^	-0.19 (-0.35,-0.04)*	-0.24 (-0.42,-0.06)*	-0.15 (-0.01,0.31)	-0.27 (-0.47,-0.06)	**0.022**	-0.39 (-0.68,-0.10)	**0.022**
*Subscapular*^§^	-0.17 (-0.31,-0.02)*	-0.24 (-0.42,-0.07)*	-0.16 (0.01,0.31)*	-0.29 (-0.49,-0.10)	**0.010**	-0.35 (-0.65,-0.04)	**0.026**
Waist circumference^§^ (cm)	-0.21 (-0.44,0.02)	-0.21 (-0.48,0.06)	-0.18 (-0.05,0.41)	-0.28 (-0.58,-0.02)	0.09	-0.45 (-0.88,-0.02)	0.07
Hip circumference^§^ (cm)	-0.34 (-0.57,-0.12)*	-0.28 (-0.54,-0.02)	-0.27 (0.05,0.50)*	-0.36 (-0.65,-0.07)	**0.034**	-0.54 (-0.95,-0.14)	**0.022**
Waist-to-hip ratio	0.001 (0, 0.003)	0 (-0.002,0.002)	0 (-0.001,0.003)	0 (-0.002,0.003)	0.73	0 (-0.002,0.004)	0.43
Arm circumference^§^ (cm)	-0.15 (-0.24,-0.06)*	-0.16 (-0.26,-0.06)*	-0.10 (0.01,0.19)*	-0.17 (-0.29,-0.06)	**0.010**	-0.22 (-0.39,-0.05)	**0.022**
**BMI categories**	**OR (95%CI)**	**OR (95%CI)**	**OR (95%CI)**	**OR (95%CI)**	***p***	**OR (95%CI)**	***p***
Thinness	1.00 (0.88–1.15)	0.98 (0.84–1.16)	1.04 (0.91–1.19)	0.95 (0.77–1.17)	0.63	1.09 (0.85–1.41)	0.49
Normal weight	1	1	1	1		1	
Overweight	0.86 (0.76–0.98)*	0.80 (0.68–0.94)*	0.91 (0.80–1.04)	0.78 (0.64–0.96)	**0.021**	0.82 (0.66–1.03)	0.09
Obese	0.92 (0.76–1.10)	0.93 (0.75–1.15)	0.92 (0.77–1.11)	0.87 (0.66–1.15)	0.34	0.75 (0.54–1.06)	0.10
*Overweight or obese*	0.87 (0.78–0.98)*	0.84 (0.74–0.96)*	0.91 (0.81–1.02)	0.81 (0.68–0.97)	**0.020**	0.81 (0.66–0.99)	**0.036**

^ SNP alleles used as independent variables;

^§^ log-transformed variables;

* nominally significant (p<0.05) genotype-phenotype associations;

bold: significant haplotype-phenotype associations for the primary phenotype (BMI, p<0.05) and for secondary variables (false discovery rate adjusted p values—pFDR <0.05); the inclusion of BMI *p* values among false discovery rate (FDR) adjustment lead to *p*FDR values of 0.017 and 0.030 (BMI and *z*-scores at T0) and 0.015 and 0.020 (at T1), with no changes in FDR statistical significance for the other variables. Detailed results for all haplotypes and for all genetic models are reported in S2 Table.

**Fig 1:**
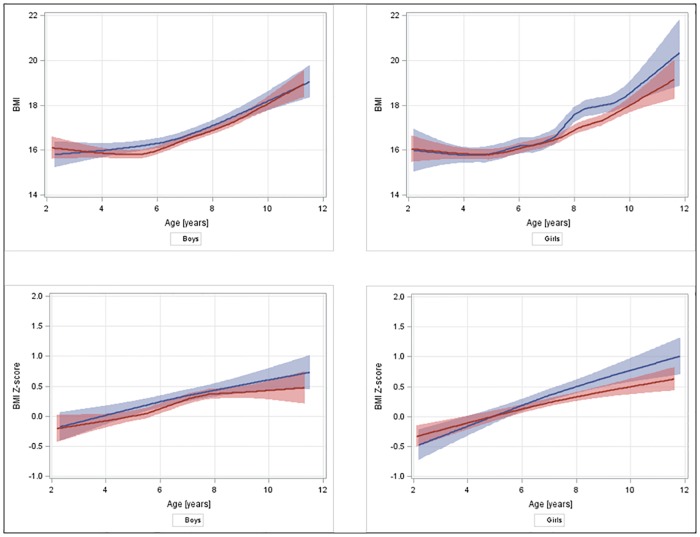
BMI (crude values and *z*-scores) age trend with 95% confidence intervals by haplotypes (CCT/x, red, vs carriers of the most prevalent haplotype, TTC, blue) in boys and girls. The graphs were drawn pooling T0 (2–10 years) and T1 (4–12 years) data to have a single comprehensive view. All children contributed with their measures weighted by haplotype posterior probabilities. Each BMI value of children contributing with both T0 and T1 measures was also weighted 0.5, to take into account the effect of repeated observations in the same subject. Local regression method implies that statistical power decreases at extreme x values (larger confidence intervals).

A significant interaction between the CCT haplotype and sex was observed only at T1 (BMI *p* = 0.02); after stratification by sex, an interaction between CCT and age was significant only in girls (*p* = 0.008). Fig 1 shows the BMI age trend by CCT haplotype status in boys and girls. In girls, a difference between curves appeared to start after 7 years and became evident between 7 and 9 years.

### Association with other anthropometric parameters

The association between anthropometric indices and genotypes were consistent with the observed associations with BMI (Table 4). Haplotype analysis confirmed CCT as the only one significantly associated with lower anthropometric values, with similar associations at T0 and T1 (Table 4 and S2 Table).

Children’s weight was lower by 0.31 (95%CI:-0.55,-0.08; *p*FDR = 0.028) and 0.59 (-0.99,-0.19; *p*FDR = 0.020) kilograms at T0 and T1, respectively, for each haplotype copy. This effect was driven by fat mass, while fat-free mass reduction was not significant. Skinfolds and hip circumference were also significantly decreased at both T0 and T1 (Table 4). Conversely, waist circumference was significantly decreased only in FDR-unadjusted analysis, while its derived measures, such as waist-to-height and waist-to-hip ratios, were not significantly modified.

## Discussion

Our study shows an association between *NMU* polymorphisms and adiposity related indices in a large population of European children, using a haplotype-based approach. The main result is the identification of a haplotype strongly associated with BMI, in a small haplotype block of the *NMU* gene. Moreover, we observed a sex specific effect and an age-dependent association in girls. Finally, we identified the anthropometric parameters which appear to drive the increase in BMI, with a stronger association with fat mass, and a minor association with measures of fat free mass.

Although considerable data from animal models are available, suggesting multiple functions for *NMU*, very few studies on humans have been performed [10,13]. Hainerovà et al. [10] identified an association between the rare homozygotes status in a missense variant of *NMU* gene and the adult overweight-obesity risk, with males showing increased waist and hip circumferences. In our study, we identified a single haplotype strongly associated with BMI and several anthropometric indices. We observed a concordant reduction in all these parameters, biologically concordant also with the reduction in bone stiffness previously observed in the same sample [13]. Our result showed in a large population that children carrying particular NMU genetic variants have lower adiposity indices and a lower risk of overweight, therefore supporting a role of *NMU* in regulating adiposity related traits.

Our approach was to use SNPs to tag a number of haplotypes potentially responsible for the effect, given the expected haplotype structure among Caucasians and the previous association observed between NMU haplotypes and bone stiffness in the same sample [13]. The absence of significant associations for the other three haplotypes confirms the utility of the haplotype approach with respect to the conventional analysis using single, intronic SNPs. This result suggests that the functional variant(s) responsible for the phenotypic variation is in linkage disequilibrium with the CCT haplotype, located in the same haplotype block or in contiguous blocks. The identification of such haplotype will allow the search for functional variant(s) through a whole-gene sequencing approach in carriers of the haplotype. In Caucasians, the CCT haplotype is in strong linkage disequilibrium with a haplotype in the first block of the gene, as reported in S1 Fig panel c (thick line in the first row). The first two haplotype blocks cover 9 out 10 exons of *NMU* and no other genes. Therefore this region includes the entire NMU-25 peptide and also the proNMU_104–136_, a longer form derived from the *NMU* gene which has shown activity similar to the shorter biologically active peptide in mice [26], as well as the cleavage region. The identification of functional variants responsible for the observed effect will be helpful in both risk assessment and treatment of metabolic diseases. Point-mutation studies demonstrated in fact that changes in amino acid sequence may alter binding of NMU to its receptors, NMUR1 and NMUR2, and may reduce or increase its half-life [27].

In GWAs, SNPs in several genes were associated with BMI, including a number of central nervous system genes as *NMU*. However they explained a very low portion of the phenotypic variance [11]. Given the strong effect as well as the high frequency of the CCT haplotype in the population (carriers 40%), *NMU* variants have instead a strong impact on BMI in children. Since higher BMI in early life increases the adult obesity risk [28], *NMU* variants could have a strong effect also in adults, for whom a drug mimicking the effect of *NMU* carrying the variants responsible for the effect could have a consistent impact on obesity reduction.

Among boys, no different effect was evident across ages. An influence of age in the association between CCT haplotype and BMI was instead evident in girls. Indeed, in girls the association between CCT and BMI appeared to be null during the adiposity rebound period and became evident starting from 7 years. In our previous analyses [13], we observed an age-dependent association in girls in the association between bone stiffness and NMU, that was most pronounced below 6 years of age. These trends could be due to age-specific expression of *NMURs* as well as *NMU* in different tissues, which was observed to vary during ageing and to be regulated by hormones such as ovarian steroids [15,29,30].

We investigated the different components of adiposity using biological impedance measurements and found that the *NMU* CCT haplotype is associated with a low fat mass. However, an association with fat-free mass is also suggested by our data, since both fat-free mass (non-significant) and arm and hip circumferences were lower in the presence of CCT haplotype. This result is in line with the previously observed negative association with bone stiffness [13] and with mouse model results [3]. The negative association with arm circumferences suggests an effect also on muscle mass, according to previous evidence of *NMU* on physical activity [1]. The reduction in fat mass as well as in hip and arm circumferences could be then explained by the following activities ascribed to NMU: decreased energy intake, through appetite regulation; improved metabolism, through insulin sensitivity modulation; increased energy expenditure, through modulation of gross-locomotor activity and heat production; decreased growth and/or activity of adipocytes, osteoblasts and myocytes [1]. The complex interplay among these mechanisms could be responsible also for the observed effect on overweight rather than in obesity. Further analyses on different phenotypes potentially involved could help to elucidate the intermediate factors responsible for the NMU-mediated effect on anthropometric measures.

The strength of this study was the possibility to evaluate in humans the effect of NMU, which is difficult to detect in plasma or serum [31]. Moreover, phenotypes in children are more heritable than those in adults, since the environment has had less time to exert its effect, therefore they represent a good model for genetic studies. Finally, the large sample size, the large number of standardized phenotypic measurements, and the availability of T1 data to check the main results in a second time point strengthen the reliability of the study. As a limitation, the sample size was not powered enough to confirm an association of CCT haplotype with waist circumference. However, this association was suggested by the significant results obtained in sensitivity analyses using dominant model or using all merged haplotypes as reference. Finally, we acknowledge that our tag SNP selection strategy covered only the haplotypes at frequency higher than 10%. However, this lead to miss the discrimination of only two haplotypes at low frequency, included in the most prevalent haplotype.

In conclusion, our data support an age- and sex-specific role of *NMU* in adiposity regulation in children. The effect was mainly driven by peripheral fat mass, although an association with indicators of bone and muscle mass was evident as well. Sequencing this short haplotypic block in carriers of CCT haplotype will allow to identify the specific DNA loci involved, useful for obesity risk assessment and, if located in the exons, for the development of new NMU analogs and inhibitors.

## Supporting information

S1 Fig NMU gene, linkage disequilibrium plot and haplotype structure. Gene coordinates: chr4: 55595229–55636698,GRCh38.p7 Assembly; Haploview v.4.2, Caucasian population (HapMap-CEU data).
(PDF)

S2 Fig Linkage disequilibrium plot of the studied haplotype block. Coordinates: 55626196–55632082 (NMU); Haploview v.4.2, Caucasian population (HapMap-CEU data). Blue arrows indicate the studied tag SNPs.
(PDF)

S1 Table Linkage disequilibrium (r^2^) between the tag SNPs rs6827359, rs12500837, rs9999653. Caucasian population (HapMap-CEU data).
(DOCX)

S2 Table Results of the association analyses between phenotypes (collected at T0, upper panel, and at T1, lower panel) and genotypes or haplotypes (most frequent haplotype—TTC—or haplotype carrying ancestral alleles—TTT—as reference). Codominant, dominant and recessive models.
(PDF)
