# Supplementary material for: The role of neuromedin U in adiposity regulation. Haplotype analysis in European children from the IDEFICS Cohort
Source: PLoS One. 2017 Feb 24;12(2):e0172698. doi: 10.1371/journal.pone.0172698 (PMC5325300; doi:10.1371/journal.pone.0172698)

S1 Fig. *NMU* gene, linkage disequilibrium plot and haplotype structure. Gene coordinates: chr4: 55595229-55636698,GRCh38.p7 Assembly; Haploview v.4.2, Caucasian population (HapMap-CEU data). Gianfagna F et al, The Role of neuromedin U in Adiposity Regulation. Haplotype Analysis in European Children from the IDEFICS Cohort; *Plos One* 2017, doi:10.1371/journal.pone.0172698

Panel a. NMU structure. Exons are indicated by full blue boxes. Single Nucleotide Polymorphisms within the studied haplotype block (red rectangle, coordinates: 55626196-55632082) are represented by black lines below the gene.
Panel b. NMU LD plot. The studied haplotype block is marked in red (enlarged version in S2 Fig). Blue arrows indicate the studied tag SNPs.
Panel c. NMU Haplotypes with frequency >10%. Blue arrows indicate the studied tag SNPs. The CCT haplotype (see text) is in high linkage with the 1st haplotype of the 1st block (black arrow)

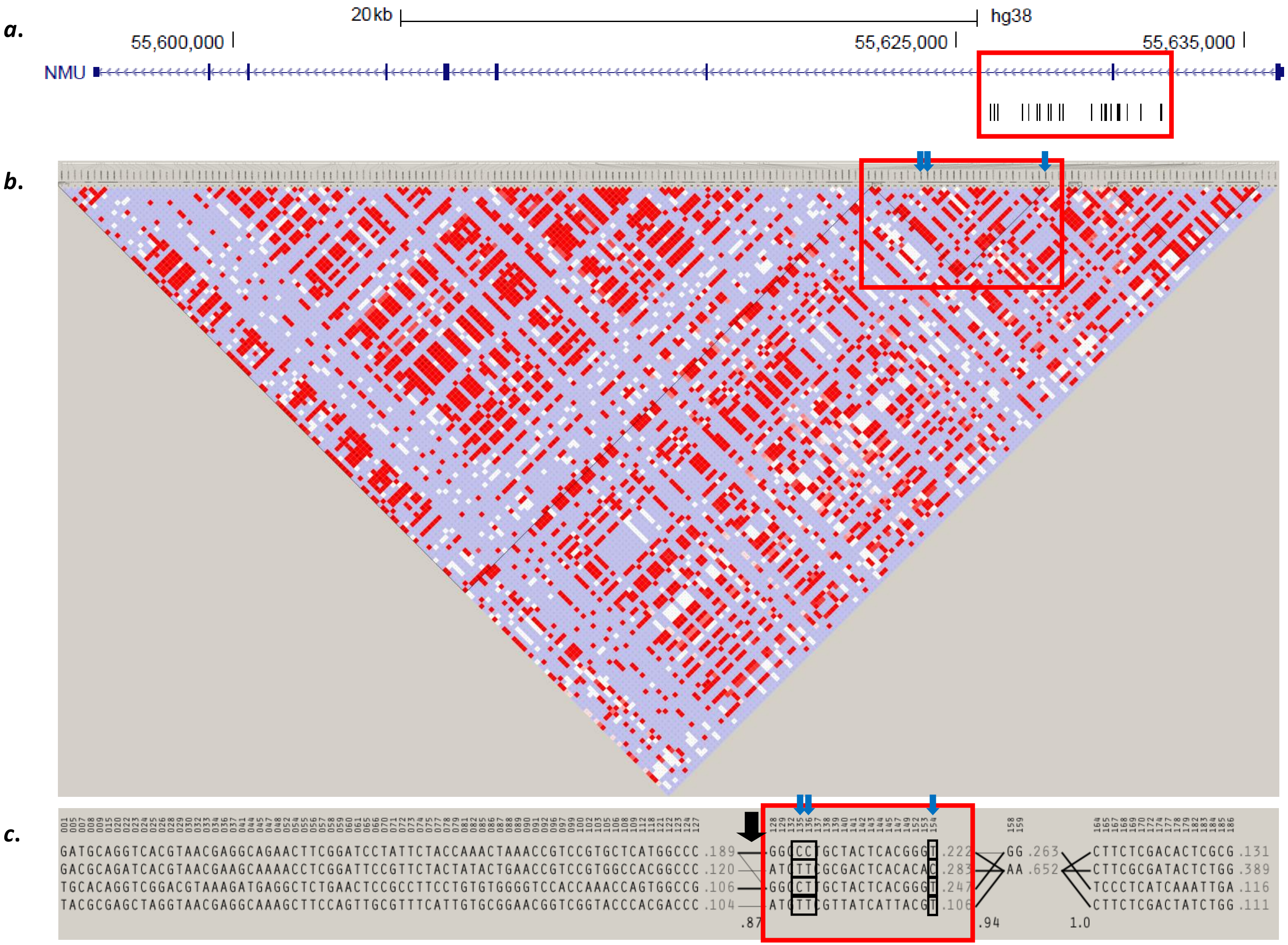

Supplement: S1 Fig — Gene coordinates: chr4: 55595229–55636698,GRCh38.p7 Assembly; Haploview v.4.2, Caucasian population (HapMap-CEU data). (PDF) [file pone.0172698.s001.pdf]
