# Supplementary material for: The role of neuromedin U in adiposity regulation. Haplotype analysis in European children from the IDEFICS Cohort
Source: PLoS One. 2017 Feb 24;12(2):e0172698. doi: 10.1371/journal.pone.0172698 (PMC5325300; doi:10.1371/journal.pone.0172698)

S2 Fig. Linkage disequilibrium plot of the studied haplotype block. Coordinates: 55626196-55632082 (*NMU*); Haploview v.4.2, Caucasian population (HapMap-CEU data). Blue arrows indicate the studied tag SNPs. Gianfagna F et al, The Role of neuromedin U in Adiposity Regulation. Haplotype Analysis in European Children from the IDEFICS Cohort; *Plos One* 2017, doi:10.1371/journal.pone.0172698

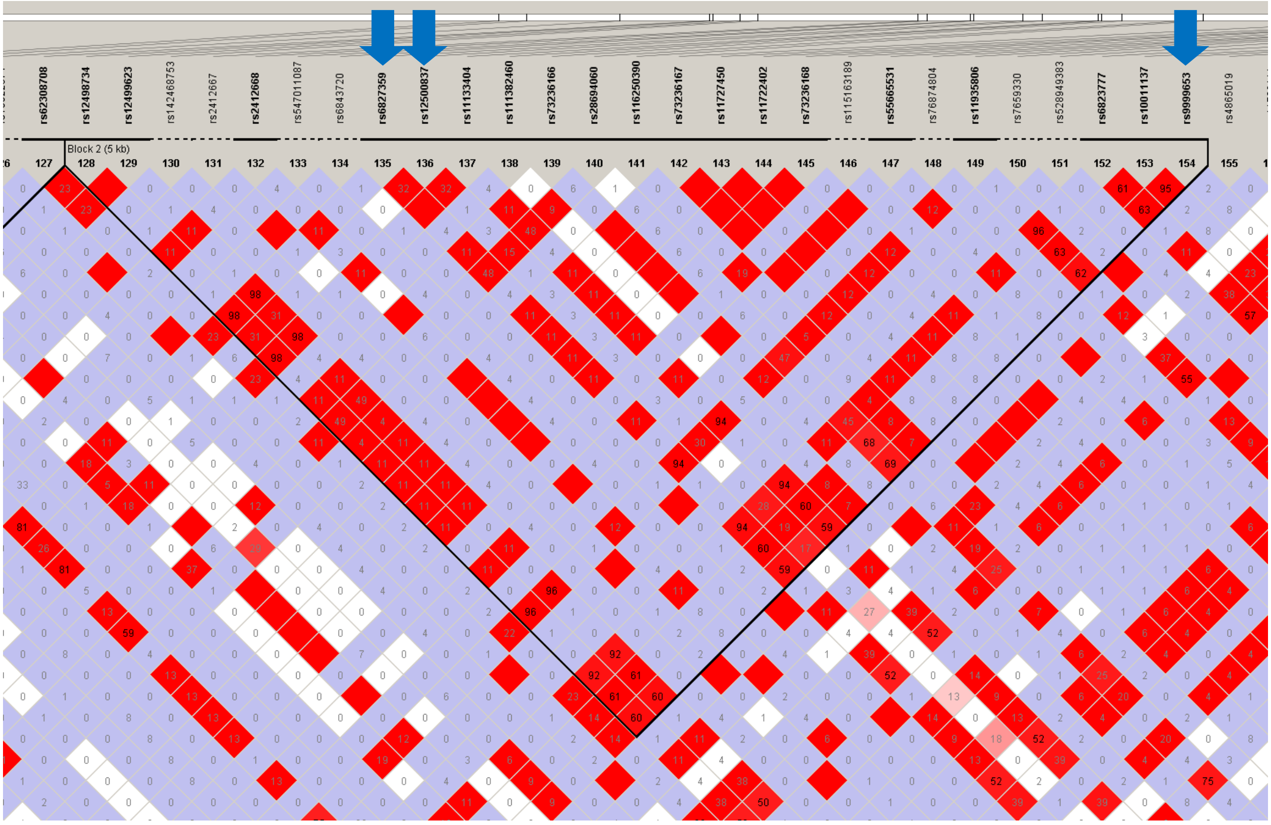

Supplement: S2 Fig — Coordinates: 55626196–55632082 (NMU); Haploview v.4.2, Caucasian population (HapMap-CEU data). Blue arrows indicate the studied tag SNPs. (PDF) [file pone.0172698.s002.pdf]
