# Supplementary material for: The role of neuromedin U in adiposity regulation. Haplotype analysis in European children from the IDEFICS Cohort
Source: PLoS One. 2017 Feb 24;12(2):e0172698. doi: 10.1371/journal.pone.0172698 (PMC5325300; doi:10.1371/journal.pone.0172698)
Supplement: S1 Table — Caucasian population (HapMap-CEU data). (DOCX) [file pone.0172698.s003.docx]

**S1 Table.** Linkage disequilibrium (r^2^) between the tag SNPs rs6827359, rs12500837, rs9999653. Caucasian population (HapMap-CEU data). Gianfagna F et al, The Role of neuromedin U in Adiposity Regulation. Haplotype Analysis in European Children from the IDEFICS Cohort; *Plos One* 2017, doi: 10.1371/journal.pone.0172698

| r^2^ | rs6827359 | rs12500837 | rs9999653 |
| --- | --- | --- | --- |
| rs6827359 | 1 | 0.32 | 0.59 |
| rs12500837 | 0.32 | 1 | 0.17 |
| rs9999653 | 0.59 | 0.17 | 1 |
