# Supplementary material for: The role of neuromedin U in adiposity regulation. Haplotype analysis in European children from the IDEFICS Cohort
Source: PLoS One. 2017 Feb 24;12(2):e0172698. doi: 10.1371/journal.pone.0172698 (PMC5325300; doi:10.1371/journal.pone.0172698)
Supplement: S2 Table — Codominant, dominant and recessive models. (PDF) [file pone.0172698.s004.pdf]

S2 Table. Results of the association analyses between phenotypes (collected at T0, upper panel, and at T1, lower panel) and genotypes or haplotypes (most frequent haplotype - TTC - or haplotype carrying ancestral alleles – TTT - as reference). Codominant, dominant and recessive models. Gianfagna F et al, The Role of neuromedin U in Adiposity Regulation. Haplotype Analysis in European Children from the IDEFICS Cohort; *Plos One* 2017, doi:10.1371/journal.pone.0172698

Codominant model		SNPs (presence vs absence)												Most frequent haplotype (TTC , 43.9%) as reference																Haplotype carrying ancestral alleles (TTT, 7.2%) as reference															
**Phenotype at T0**		rs6827359 C (48.7%)				rs12500837 C (24.4%)				rs9999653 T (53.9%)				CCC (2.0%)				CCT (22.3%)				CTT (24.4%)				TTT (7.2%)				CCC (2.0%)				CCT (22.3%)				CTT (24.4%)				TTC (43.9%)			
	N	beta		se	p	beta		se	p	beta		se	p	beta		se	p	beta		se	p	beta		se	p	beta		se	p	beta		se	p	beta		se	p	beta		se	p	beta		se	p
Bmi (ln)	4528	-0.12	±	0.05	0.006	-0.13	±	0.05	0.012	-0.10	±	0.05	0.031	-0.05	±	0.18	0.80	-0.16	±	0.06	0.006	-0.08	±	0.06	0.15	0.06	±	0.10	0.52	-0.11	±	0.19	0.58	-0.22	±	0.10	0.026	-0.14	±	0.10	0.14	-0.06	±	0.10	0.53
Bmi z-score	4528	-0.07	±	0.03	0.010	-0.06	±	0.03	0.040	-0.05	±	0.03	0.042	0.00	±	0.10	1.00	-0.08	±	0.03	0.019	-0.05	±	0.03	0.12	0.04	±	0.05	0.44	-0.04	±	0.11	0.70	-0.12	±	0.06	0.034	-0.09	±	0.06	0.10	-0.04	±	0.05	0.45
Weight, Kg (ln)	4528	-0.26	±	0.09	0.007	-0.27	±	0.11	0.026	-0.18	±	0.09	0.06	-0.17	±	0.35	0.74	-0.31	±	0.12	0.017	-0.18	±	0.12	0.13	0.19	±	0.19	0.29	-0.35	±	0.38	0.41	-0.50	±	0.20	0.017	-0.36	±	0.20	0.06	-0.18	±	0.19	0.32
Fat mass, %	4463	-0.17	±	0.06	0.004	-0.15	±	0.07	0.031	-0.14	±	0.06	0.019	-0.08	±	0.23	0.72	-0.20	±	0.08	0.011	-0.15	±	0.08	0.06	0.05	±	0.13	0.68	-0.14	±	0.25	0.59	-0.26	±	0.13	0.05	-0.20	±	0.13	0.13	-0.05	±	0.13	0.68
Fat-free mass, %	4454	-0.08	±	0.05	0.09	-0.10	±	0.05	0.08	-0.03	±	0.05	0.50	-0.07	±	0.18	0.68	-0.09	±	0.06	0.14	-0.03	±	0.06	0.59	0.16	±	0.10	0.11	-0.23	±	0.19	0.23	-0.24	±	0.10	0.017	-0.18	±	0.10	0.07	-0.15	±	0.10	0.12
Tricipital+subscapular skinfolds, mm (ln)	4,391	-0.33	±	0.15	0.015	-0.48	±	0.17	0.005	-0.30	±	0.15	0.036	-0.43	±	0.55	0.35	-0.55	±	0.19	0.004	-0.17	±	0.19	0.30	-0.09	±	0.30	0.89	-0.35	±	0.60	0.43	-0.47	±	0.32	0.11	-0.08	±	0.32	0.64	0.09	±	0.30	0.88
Tricipital skinfold, mm (ln)	4416	-0.19	±	0.08	0.017	-0.24	±	0.09	0.011	-0.15	±	0.08	0.07	-0.37	±	0.30	0.24	-0.27	±	0.10	0.011	-0.11	±	0.10	0.28	0.01	±	0.16	0.88	-0.39	±	0.32	0.25	-0.28	±	0.17	0.10	-0.13	±	0.17	0.43	-0.01	±	0.16	0.87
Subscapular skinfold, mm (ln)	4413	-0.17	±	0.08	0.016	-0.24	±	0.09	0.005	-0.16	±	0.08	0.020	-0.09	±	0.29	0.54	-0.29	±	0.10	0.002	-0.08	±	0.10	0.28	-0.06	±	0.16	0.65	-0.03	±	0.31	0.75	-0.23	±	0.17	0.17	-0.02	±	0.16	0.88	0.06	±	0.16	0.61
waist circumference, cm (ln)	4527	-0.21	±	0.12	0.08	-0.21	±	0.14	0.12	-0.18	±	0.12	0.14	0.20	±	0.45	0.66	-0.28	±	0.15	0.07	-0.15	±	0.15	0.36	0.14	±	0.24	0.52	0.07	±	0.48	0.92	-0.41	±	0.26	0.10	-0.28	±	0.25	0.26	-0.13	±	0.24	0.55
hip circircumference, cm (ln)	4527	-0.34	±	0.11	0.003	-0.28	±	0.13	0.06	-0.27	±	0.11	0.022	-0.40	±	0.43	0.39	-0.36	±	0.15	0.024	-0.30	±	0.14	0.032	0.08	±	0.23	0.69	-0.47	±	0.46	0.33	-0.43	±	0.25	0.10	-0.37	±	0.24	0.12	-0.06	±	0.23	0.76
waist-to-hip ratio	4526	0.001	±	0.001	0.17	0.000	±	0.001	0.77	0.001	±	0.001	0.43	0.008	±	0.004	0.031	0.000	±	0.001	0.74	0.002	±	0.001	0.15	0.001	±	0.002	0.59	0.007	±	0.004	0.08	-0.001	±	0.002	0.73	0.001	±	0.002	0.77	-0.001	±	0.002	0.56
arm circumference, cm (ln)	4497	-0.15	±	0.04	0.001	-0.16	±	0.05	0.002	-0.10	±	0.04	0.022	-0.18	±	0.17	0.32	-0.17	±	0.06	0.002	-0.10	±	0.06	0.07	0.11	±	0.09	0.25	-0.28	±	0.18	0.14	-0.27	±	0.10	0.005	-0.20	±	0.09	0.037	-0.10	±	0.09	0.28
ref: normal weight (N=3234)		OR	95%CI		p	OR	95%CI		p	OR	95%CI		p	OR	95%CI		p	OR	95%CI		p	OR	95%CI		p	OR	95%CI		p	OR	95%CI		p	OR	95%CI		p	OR	95%CI		p	OR	95%CI		p
Thinness	478	1.00	0.88	1.15	0.97	0.99	0.84	1.16	0.85	1.04	0.91	1.19	0.57	0.64	0.36	1.16	0.14	0.95	0.77	1.17	0.63	1.01	0.82	1.24	0.94	0.96	0.69	1.32	0.79	0.38	0.12	1.28	0.12	0.81	0.52	1.27	0.36	1.11	0.75	1.64	0.60	1.05	0.82	1.35	0.70
Overweight	541	0.86	0.76	0.98	0.028	0.80	0.68	0.94	0.006	0.91	0.80	1.04	0.17	0.59	0.34	1.03	0.06	0.78	0.64	0.96	0.021	0.88	0.73	1.07	0.20	0.95	0.69	1.30	0.74	0.23	0.07	0.80	0.021	0.71	0.45	1.13	0.15	0.75	0.50	1.13	0.17	1.12	0.88	1.41	0.36
Obese	275	0.92	0.76	1.10	0.35	0.93	0.75	1.15	0.52	0.92	0.77	1.11	0.39	0.93	0.47	1.82	0.82	0.87	0.66	1.15	0.34	0.94	0.72	1.24	0.67	0.94	0.59	1.49	0.78	0.17	0.03	0.92	0.040	0.67	0.36	1.27	0.22	0.98	0.56	1.70	0.93	1.00	0.72	1.39	0.99
Overweight or obese	816	0.88	0.78	0.98	0.021	0.84	0.74	0.97	0.013	0.91	0.81	1.02	0.11	0.69	0.44	1.09	0.11	0.81	0.68	0.97	0.020	0.89	0.75	1.06	0.18	0.95	0.73	1.25	0.73	0.19	0.06	0.59	0.004	0.70	0.48	1.03	0.07	0.82	0.58	1.15	0.25	1.07	0.87	1.31	0.51

**Phenotype at T1**		rs6827359 C (48.2%)				rs12500837 C (24.4%)				rs9999653 T (53.9%)				CCC (2.0%)				CCT (22.0%)				CTT (24.4%)				TTT (7.4%)				CCC (2.0%)				CCT (22.0%)				CTT (24.4%)				TTC (44.0%)			
		beta		se	p	beta		se	p	beta		se	p	beta		se	p	beta		se	p	beta		se	p	beta		se	p	beta		se	p	beta		se	p	beta		se	p	beta		se	p
Bmi (ln)	3277	-0.16	±	0.06	0.021	-0.24	±	0.08	0.001	-0.10	±	0.07	0.13	-0.30	±	0.24	0.24	-0.25	±	0.08	0.004	-0.06	±	0.08	0.62	0.07	±	0.13	0.63	-0.36	±	0.26	0.18	-0.31	±	0.14	0.029	-0.12	±	0.14	0.47	-0.06	±	0.13	0.66
Bmi z-score	3277	-0.05	±	0.03	0.07	-0.11	±	0.04	0.003	-0.03	±	0.03	0.25	-0.10	±	0.11	0.38	-0.10	±	0.04	0.009	0.00	±	0.04	0.97	0.03	±	0.06	0.61	-0.13	±	0.12	0.29	-0.13	±	0.07	0.042	-0.03	±	0.06	0.62	-0.03	±	0.06	0.63
Weight, Kg (ln)	3277	-0.41	±	0.15	0.018	-0.61	±	0.18	0.001	-0.23	±	0.15	0.18	-0.95	±	0.58	0.13	-0.59	±	0.20	0.004	-0.15	±	0.19	0.69	0.27	±	0.31	0.45	-1.21	±	0.62	0.08	-0.85	±	0.33	0.015	-0.41	±	0.33	0.35	-0.26	±	0.31	0.48
Fat mass, %	3227	-0.33	±	0.10	0.002	-0.43	±	0.12	0.001	-0.24	±	0.11	0.024	-0.74	±	0.39	0.06	-0.46	±	0.14	0.001	-0.19	±	0.13	0.16	0.02	±	0.21	0.94	-0.75	±	0.42	0.08	-0.47	±	0.23	0.037	-0.20	±	0.22	0.37	-0.01	±	0.21	0.96
Fat-free mass, %	3227	-0.10	±	0.07	0.14	-0.20	±	0.08	0.015	-0.04	±	0.07	0.60	-0.19	±	0.27	0.48	-0.18	±	0.09	0.06	0.01	±	0.09	0.90	0.17	±	0.14	0.24	-0.36	±	0.28	0.21	-0.34	±	0.15	0.025	-0.15	±	0.15	0.30	-0.16	±	0.14	0.25
Tric.+subsc. Skinfolds, mm (ln)	3180	-0.45	±	0.22	0.08	-0.66	±	0.26	0.006	-0.37	±	0.22	0.18	-1.01	±	0.82	0.12	-0.72	±	0.29	0.013	-0.24	±	0.28	0.71	-0.10	±	0.45	0.76	-0.91	±	0.89	0.20	-0.63	±	0.48	0.22	-0.15	±	0.47	0.96	0.09	±	0.45	0.78
Tricipital skinfold, mm (ln)	3184	-0.26	±	0.11	0.07	-0.39	±	0.13	0.003	-0.19	±	0.11	0.21	-0.91	±	0.42	0.029	-0.39	±	0.15	0.013	-0.14	±	0.14	0.73	-0.09	±	0.23	0.75	-0.84	±	0.45	0.06	-0.32	±	0.24	0.21	-0.06	±	0.24	0.95	0.07	±	0.23	0.78
Subscapular skinfold, mm (ln)	3183	-0.21	±	0.12	0.11	-0.29	±	0.14	0.015	-0.19	±	0.12	0.16	-0.11	±	0.45	0.41	-0.35	±	0.16	0.018	-0.12	±	0.15	0.69	-0.04	±	0.24	0.72	-0.07	±	0.48	0.55	-0.31	±	0.26	0.27	-0.08	±	0.25	0.92	0.04	±	0.24	0.73
waist circumference, cm (ln)	3222	-0.28	±	0.17	0.13	-0.47	±	0.20	0.021	-0.15	±	0.17	0.46	-0.65	±	0.63	0.30	-0.45	±	0.22	0.05	-0.06	±	0.21	0.86	0.24	±	0.34	0.47	-0.89	±	0.67	0.19	-0.69	±	0.36	0.06	-0.30	±	0.35	0.43	-0.24	±	0.34	0.47
hip circircumference, cm (ln)	3205	-0.36	±	0.16	0.038	-0.56	±	0.19	0.003	-0.22	±	0.16	0.21	-0.97	±	0.59	0.13	-0.54	±	0.21	0.011	-0.12	±	0.20	0.68	0.14	±	0.32	0.69	-1.09	±	0.64	0.11	-0.66	±	0.34	0.06	-0.25	±	0.33	0.57	-0.12	±	0.32	0.74
waist-to-hip ratio	3200	0.001	±	0.001	0.32	0.001	±	0.001	0.62	0.001	±	0.001	0.23	0.001	±	0.004	0.80	0.001	±	0.002	0.43	0.001	±	0.001	0.33	0.002	±	0.002	0.37	-0.001	±	0.005	0.79	-0.001	±	0.003	0.65	-0.001	±	0.002	0.71	-0.002	±	0.002	0.31
arm circumference, cm (ln)	3016	-0.20	±	0.06	0.003	-0.21	±	0.08	0.007	-0.12	±	0.07	0.07	-0.39	±	0.25	0.15	-0.22	±	0.09	0.009	-0.13	±	0.08	0.13	0.12	±	0.13	0.39	-0.51	±	0.27	0.08	-0.34	±	0.14	0.018	-0.25	±	0.14	0.09	-0.12	±	0.13	0.41
ref: normal weight (N=2256)		OR	95%CI		p	OR	95%CI		p	OR	95%CI		p	OR	95%CI		p	OR	95%CI		p	OR	95%CI		p	OR	95%CI		p	OR	95%CI		p	OR	95%CI		p	OR	95%CI		p	OR	95%CI		p
Thinness	324	1.03	0.87	1.21	0.76	1.12	0.93	1.36	0.24	1.07	0.90	1.26	0.43	0.48	0.22	1.03	0.06	1.09	0.85	1.41	0.49	0.97	0.75	1.25	0.82	0.88	0.60	1.29	0.52	0.41	0.10	1.67	0.21	1.36	0.84	2.18	0.21	1.52	0.96	2.43	0.08	1.18	0.87	1.60	0.28
Overweight	496	0.88	0.76	1.01	0.07	0.82	0.69	0.97	0.021	0.92	0.80	1.06	0.26	0.53	0.29	0.99	0.046	0.82	0.66	1.03	0.09	0.94	0.76	1.17	0.59	0.91	0.65	1.28	0.60	1.03	0.18	5.99	0.98	0.85	0.52	1.39	0.51	1.23	0.82	1.86	0.32	1.09	0.85	1.41	0.50
Obese	201	0.84	0.68	1.04	0.11	0.80	0.61	1.03	0.09	0.85	0.68	1.05	0.13	0.69	0.32	1.51	0.36	0.75	0.54	1.06	0.10	0.86	0.63	1.19	0.36	0.80	0.47	1.37	0.42	0.34	0.06	2.08	0.24	1.17	0.58	2.38	0.66	1.04	0.55	1.98	0.89	1.27	0.87	1.85	0.22
Overweight or obese	697	0.87	0.76	0.98	0.025	0.82	0.70	0.95	0.010	0.90	0.79	1.02	0.10	0.59	0.35	0.98	0.042	0.81	0.66	0.99	0.036	0.91	0.76	1.10	0.33	0.89	0.65	1.20	0.43	0.70	0.19	2.66	0.60	0.96	0.63	1.46	0.84	1.18	0.82	1.71	0.37	1.14	0.91	1.43	0.25

(cont.)

Dominant model

Phenotype at T0 — SNPs (presence vs absence)

Phenotype at T0	N	rs6827359 C (73.1%) beta	se	p	rs12500837 C (42.7%) beta	se	p	rs9999653 T (78.5%) beta	se	p
Bmi (ln)	4528	-0.15	± 0.07	0.043	-0.17	± 0.07	0.013	-0.07	± 0.07	0.012
Bmi z-score	4528	-0.06	± 0.04	0.12	-0.08	± 0.04	0.041	-0.03	± 0.04	0.013
Weight, Kg (ln)	4528	-0.35	± 0.15	0.044	-0.34	± 0.13	0.016	-0.15	± 0.15	0.024
Fat mass, %	4463	-0.25	± 0.10	0.013	-0.19	± 0.09	0.034	-0.13	± 0.10	0.011
Fat-free mass, %	4454	-0.10	± 0.07	0.18	-0.13	± 0.07	0.05	-0.02	± 0.07	0.39
Tricipital+subscapular skinfolds, mm (ln)	4,391	-0.49	± 0.24	0.029	-0.56	± 0.21	0.010	-0.30	± 0.23	0.039
Tricipital skinfold, mm (ln)	4416	-0.31	± 0.13	0.027	-0.27	± 0.11	0.024	-0.19	± 0.12	0.10
Subscapular skinfold, mm (ln)	4413	-0.24	± 0.12	0.024	-0.31	± 0.11	0.006	-0.16	± 0.12	0.019
waist circumference, cm (ln)	4527	-0.28	± 0.19	0.18	-0.31	± 0.17	0.06	-0.11	± 0.18	0.06
hip circircumference, cm (ln)	4527	-0.48	± 0.18	0.015	-0.39	± 0.16	0.023	-0.32	± 0.18	0.028
waist-to-hip ratio	4526	0.002	± 0.002	0.21	0.000	± 0.001	0.83	0.003	± 0.002	0.91
arm circumference, cm (ln)	4497	-0.22	± 0.07	0.002	-0.20	± 0.06	0.002	-0.13	± 0.07	0.044
ref: normal weight (N=3234)		**OR**	**95%CI**	**p**	**OR**	**95%CI**	**p**	**OR**	**95%CI**	**p**
Thinness	478	0.91	0.74 1.14	0.42	1.00	0.82 1.22	1.00	0.98	0.77 1.24	0.30
Overweight	541	0.79	0.65 0.97	0.027	0.77	0.63 0.93	0.006	0.87	0.70 1.09	0.29
Obese	275	0.83	0.62 1.11	0.20	0.94	0.72 1.22	0.62	0.94	0.69 1.28	0.34
Overweight or obese	816	0.80	0.67 0.96	0.015	0.82	0.70 0.97	0.018	0.89	0.74 1.08	0.15

Phenotype at T0 — Most frequent haplotype (TTC , 43.9%) as reference

Phenotype at T0	N	CCC/x (4.0%) beta	se	p	CCT/x (39.7%) beta	se	p	CTT/x (42.5%) beta	se	p	TTT/x (13.8%) beta	se	p
Bmi (ln)	4528	-0.04	± 0.18	0.84	-0.18	± 0.07	0.009	-0.08	± 0.07	0.23	0.03	± 0.10	0.78
Bmi z-score	4528	0.00	± 0.10	0.96	-0.09	± 0.04	0.026	-0.05	± 0.04	0.21	0.03	± 0.06	0.65
Weight, Kg (ln)	4528	-0.13	± 0.36	0.82	-0.34	± 0.14	0.021	-0.16	± 0.14	0.25	0.18	± 0.20	0.34
Fat mass, %	4463	-0.06	± 0.23	0.79	-0.21	± 0.09	0.022	-0.15	± 0.09	0.11	0.01	± 0.13	0.93
Fat-free mass, %	4454	-0.06	± 0.18	0.74	-0.11	± 0.07	0.13	-0.02	± 0.07	0.74	0.18	± 0.10	0.08
Tricipital+subscapular skinfolds, mm (ln)	4,391	-0.41	± 0.56	0.37	-0.61	± 0.23	0.007	-0.18	± 0.22	0.30	-0.22	± 0.32	0.57
Tricipital skinfold, mm (ln)	4416	-0.36	± 0.30	0.25	-0.29	± 0.12	0.017	-0.14	± 0.12	0.25	-0.06	± 0.17	0.76
Subscapular skinfold, mm (ln)	4413	-0.08	± 0.29	0.54	-0.34	± 0.12	0.003	-0.09	± 0.12	0.25	-0.15	± 0.17	0.32
waist circumference, cm (ln)	4527	0.22	± 0.45	0.64	-0.35	± 0.18	0.045	-0.16	± 0.18	0.41	0.06	± 0.26	0.76
hip circircumference, cm (ln)	4527	-0.38	± 0.43	0.40	-0.44	± 0.17	0.017	-0.32	± 0.17	0.06	0.03	± 0.25	0.88
waist-to-hip ratio	4526	0.009	± 0.004	0.028	0.000	± 0.002	0.81	0.002	± 0.002	0.18	0.001	± 0.002	0.73
arm circumference, cm (ln)	4497	-0.17	± 0.17	0.37	-0.20	± 0.07	0.003	-0.09	± 0.07	0.17	0.09	± 0.10	0.37
ref: normal weight (N=3234)		**OR**	**95%CI**	**p**	**OR**	**95%CI**	**p**	**OR**	**95%CI**	**p**	**OR**	**95%CI**	**p**
Thinness	478	0.61	0.27 1.39	0.24	0.91	0.68 1.22	0.52	0.94	0.70 1.25	0.66	0.95	0.63 1.44	0.81
Overweight	541	0.50	0.22 1.13	0.10	0.71	0.54 0.94	0.017	0.91	0.70 1.19	0.51	0.96	0.63 1.44	0.83
Obese	275	0.60	0.20 1.79	0.36	0.93	0.64 1.35	0.70	0.90	0.62 1.32	0.59	1.13	0.63 2.03	0.69
Overweight or obese	816	0.53	0.27 1.05	0.07	0.78	0.62 0.99	0.043	0.90	0.72 1.14	0.39	1.01	0.71 1.44	0.97

Phenotype at T0 — Haplotype carrying ancestral alleles (TTT, 7.2%) as reference*

Phenotype at T0	N	CCC/x (4.0%) beta	se	p	CCT/x (39.7%) beta	se	p	CTT/x (42.5%) beta	se	p	TTC/x (68.4%) beta	se	p
Bmi (ln)	4528	-0.02	± 0.18	0.93	-0.15	± 0.08	0.06	-0.05	± 0.08	0.51	0.07	± 0.09	0.35
Bmi z-score	4528	0.02	± 0.10	0.88	-0.07	± 0.04	0.13	-0.03	± 0.04	0.50	0.05	± 0.05	0.31
Weight, Kg (ln)	4528	-0.15	± 0.36	0.78	-0.33	± 0.16	0.048	-0.16	± 0.16	0.33	0.07	± 0.18	0.67
Fat mass, %	4463	-0.04	± 0.23	0.88	-0.17	± 0.11	0.11	-0.10	± 0.11	0.33	0.10	± 0.12	0.39
Fat-free mass, %	4454	-0.11	± 0.18	0.52	-0.16	± 0.08	0.05	-0.07	± 0.08	0.36	-0.06	± 0.09	0.53
Tricipital+subscapular skinfolds, mm (ln)	4,391	-0.28	± 0.56	0.49	-0.46	± 0.25	0.06	-0.03	± 0.25	0.68	0.26	± 0.28	0.42
Tricipital skinfold, mm (ln)	4416	-0.33	± 0.30	0.30	-0.26	± 0.14	0.07	-0.10	± 0.14	0.46	0.06	± 0.15	0.67
Subscapular skinfold, mm (ln)	4413	0.00	± 0.29	0.73	-0.26	± 0.13	0.045	0.00	± 0.13	0.72	0.14	± 0.15	0.34
waist circumference, cm (ln)	4527	0.22	± 0.45	0.64	-0.33	± 0.20	0.10	-0.13	± 0.20	0.53	0.07	± 0.23	0.76
hip circircumference, cm (ln)	4527	-0.36	± 0.43	0.43	-0.40	± 0.20	0.05	-0.28	± 0.20	0.14	0.09	± 0.22	0.70
waist-to-hip ratio	4526	0.008	± 0.004	0.032	0.000	± 0.002	0.89	0.002	± 0.002	0.27	0.000	± 0.002	0.97
arm circumference, cm (ln)	4497	-0.18	± 0.17	0.33	-0.21	± 0.08	0.007	-0.10	± 0.08	0.20	0.01	± 0.08	0.88
ref: normal weight (N=3234)		**OR**	**95%CI**	**p**	**OR**	**95%CI**	**p**	**OR**	**95%CI**	**p**	**OR**	**95%CI**	**p**
Thinness	478	0.52	0.05 5.58	0.58	0.42	0.14 1.25	0.12	0.43	0.15 1.28	0.13	0.51	0.18 1.44	0.20
Overweight	541	0.32	0.03 4.17	0.39	0.26	0.09 0.75	0.013	0.28	0.10 0.82	0.020	0.38	0.14 1.03	0.06
Obese	275	0.07	<0.001 6.02	0.24	0.16	0.03 0.77	0.023	0.18	0.04 0.82	0.027	0.29	0.08 1.10	0.07
Overweight or obese	816	0.22	0.02 1.94	0.17	0.23	0.09 0.58	0.002	0.26	0.10 0.66	0.005	0.35	0.15 0.85	0.020

Phenotype at T1 — SNPs (presence vs absence)

Phenotype at T1	N	rs6827359 C (72.4%) beta	se	p	rs12500837 C (42.3%) beta	se	p	rs9999653 T (78.0%) beta	se	p
Bmi (ln)	3277	-0.20	± 0.10	0.09	-0.26	± 0.09	0.004	-0.12	± 0.10	0.14
Bmi z-score	3277	-0.05	± 0.05	0.30	-0.11	± 0.04	0.009	-0.04	± 0.05	0.27
Weight, Kg (ln)	3277	-0.56	± 0.25	0.08	-0.69	± 0.22	0.001	-0.24	± 0.24	0.16
Fat mass, %	3227	-0.46	± 0.17	0.006	-0.49	± 0.15	0.001	-0.32	± 0.16	0.05
Fat-free mass, %	3227	-0.15	± 0.11	0.17	-0.22	± 0.10	0.033	-0.03	± 0.11	0.54
Tric.+subsc. Skinfolds, mm (ln)	3180	-0.66	± 0.35	0.21	-0.75	± 0.32	0.013	-0.42	± 0.35	0.16
Tricipital skinfold, mm (ln)	3184	-0.33	± 0.18	0.26	-0.41	± 0.16	0.010	-0.20	± 0.18	0.17
Subscapular skinfold, mm (ln)	3183	-0.35	± 0.19	0.19	-0.35	± 0.17	0.025	-0.24	± 0.19	0.18
waist circumference, cm (ln)	3222	-0.30	± 0.27	0.38	-0.56	± 0.24	0.027	-0.03	± 0.26	0.22
hip circircumference, cm (ln)	3205	-0.48	± 0.25	0.11	-0.70	± 0.23	0.002	-0.23	± 0.25	0.20
waist-to-hip ratio	3200	0.002	± 0.002	0.19	0.001	± 0.002	0.43	0.004	± 0.002	0.79
arm circumference, cm (ln)	3016	-0.28	± 0.10	0.014	-0.23	± 0.09	0.014	-0.18	± 0.10	0.13
ref: normal weight (N=2256)		**OR**	**95%CI**	**p**	**OR**	**95%CI**	**p**	**OR**	**95%CI**	**p**
Thinness	324	0.91	0.70 1.18	0.48	1.07	0.84 1.35	0.60	0.98	0.74 1.31	0.19
Overweight	496	0.85	0.68 1.06	0.14	0.81	0.66 0.99	0.041	0.93	0.73 1.18	0.24
Obese	201	0.75	0.54 1.06	0.10	0.72	0.52 0.98	0.039	0.79	0.56 1.13	0.24
Overweight or obese	697	0.82	0.67 1.00	0.048	0.79	0.66 0.95	0.011	0.88	0.71 1.09	0.13

Phenotype at T1 — Most frequent haplotype (TTC , 43.9%) as reference

Phenotype at T1	N	CCC/x (4.1%) beta	se	p	CCT/x (39.2%) beta	se	p	CTT/x (42.6%) beta	se	p	TTT/x (14.1%) beta	se	p
Bmi (ln)	3277	-0.27	± 0.25	0.30	-0.27	± 0.10	0.007	-0.03	± 0.10	0.94	0.04	± 0.14	0.72
Bmi z-score	3277	-0.08	± 0.11	0.47	-0.11	± 0.05	0.013	0.02	± 0.05	0.62	0.03	± 0.07	0.64
Weight, Kg (ln)	3277	-0.87	± 0.59	0.18	-0.64	± 0.24	0.005	-0.07	± 0.23	0.90	0.30	± 0.34	0.40
Fat mass, %	3227	-0.70	± 0.40	0.08	-0.49	± 0.16	0.002	-0.18	± 0.16	0.26	-0.02	± 0.23	0.91
Fat-free mass, %	3227	-0.14	± 0.27	0.60	-0.19	± 0.11	0.08	0.09	± 0.11	0.42	0.23	± 0.15	0.13
Tric.+subsc. Skinfolds, mm (ln)	3180	-0.95	± 0.83	0.15	-0.82	± 0.34	0.017	-0.27	± 0.33	0.90	-0.26	± 0.48	0.59
Tricipital skinfold, mm (ln)	3184	-0.84	± 0.42	0.05	-0.40	± 0.17	0.024	-0.09	± 0.17	0.86	-0.14	± 0.24	0.64
Subscapular skinfold, mm (ln)	3183	-0.12	± 0.45	0.39	-0.43	± 0.18	0.017	-0.19	± 0.18	0.62	-0.15	± 0.26	0.52
waist circumference, cm (ln)	3222	-0.62	± 0.63	0.33	-0.54	± 0.26	0.05	-0.07	± 0.25	0.93	0.17	± 0.36	0.62
hip circircumference, cm (ln)	3205	-0.90	± 0.60	0.17	-0.67	± 0.24	0.006	-0.04	± 0.24	0.94	0.13	± 0.34	0.73
waist-to-hip ratio	3200	0.000	± 0.004	0.92	0.002	± 0.002	0.38	0.000	± 0.002	0.84	0.001	± 0.003	0.63
arm circumference, cm (ln)	3016	-0.35	± 0.25	0.22	-0.23	± 0.10	0.021	-0.10	± 0.10	0.41	0.12	± 0.14	0.39
ref: normal weight (N=2256)		**OR**	**95%CI**	**p**	**OR**	**95%CI**	**p**	**OR**	**95%CI**	**p**	**OR**	**95%CI**	**p**
Thinness	324	0.23	0.06 0.99	0.048	1.06	0.75 1.49	0.76	0.82	0.58 1.17	0.28	0.82	0.49 1.36	0.44
Overweight	496	0.56	0.24 1.31	0.18	0.85	0.63 1.16	0.31	0.96	0.72 1.29	0.80	1.05	0.67 1.65	0.82
Obese	201	0.48	0.14 1.70	0.25	0.73	0.47 1.13	0.15	0.87	0.57 1.33	0.52	0.79	0.38 1.64	0.52
Overweight or obese	697	0.55	0.26 1.15	0.11	0.82	0.63 1.07	0.14	0.92	0.71 1.19	0.53	0.98	0.65 1.46	0.91

Phenotype at T1 — Haplotype carrying ancestral alleles (TTT, 7.2%) as reference*

Phenotype at T1	N	CCC/x (4.1%) beta	se	p	CCT/x (39.2%) beta	se	p	CTT/x (42.6%) beta	se	p	TTC/x (68.1%) beta	se	p
Bmi (ln)	3277	-0.26	± 0.25	0.33	-0.24	± 0.11	0.033	-0.01	± 0.11	0.85	0.07	± 0.12	0.52
Bmi z-score	3277	-0.08	± 0.12	0.50	-0.10	± 0.05	0.05	0.04	± 0.05	0.50	0.04	± 0.06	0.52
Weight, Kg (ln)	3277	-0.92	± 0.59	0.16	-0.65	± 0.27	0.015	-0.09	± 0.26	0.91	0.06	± 0.30	0.75
Fat mass, %	3227	-0.65	± 0.40	0.10	-0.43	± 0.18	0.018	-0.12	± 0.18	0.51	0.13	± 0.20	0.51
Fat-free mass, %	3227	-0.21	± 0.27	0.43	-0.25	± 0.12	0.043	0.03	± 0.12	0.83	-0.06	± 0.13	0.65
Tric.+subsc. Skinfolds, mm (ln)	3180	-0.83	± 0.84	0.20	-0.69	± 0.38	0.10	-0.15	± 0.38	0.74	0.20	± 0.42	0.49
Tricipital skinfold, mm (ln)	3184	-0.75	± 0.43	0.08	-0.29	± 0.20	0.16	0.02	± 0.19	0.46	0.21	± 0.22	0.31
Subscapular skinfold, mm (ln)	3183	-0.09	± 0.46	0.47	-0.41	± 0.21	0.07	-0.17	± 0.20	0.88	0.00	± 0.23	0.73
waist circumference, cm (ln)	3222	-0.62	± 0.64	0.34	-0.50	± 0.29	0.12	-0.03	± 0.29	0.92	0.14	± 0.32	0.60
hip circircumference, cm (ln)	3205	-0.90	± 0.60	0.17	-0.65	± 0.27	0.019	-0.02	± 0.27	0.89	0.08	± 0.30	0.80
waist-to-hip ratio	3200	0.000	± 0.004	0.94	0.002	± 0.002	0.42	0.000	± 0.002	0.84	0.000	± 0.002	0.83
arm circumference, cm (ln)	3016	-0.37	± 0.25	0.20	-0.23	± 0.11	0.048	-0.11	± 0.11	0.48	0.03	± 0.13	0.72
ref: normal weight (N=2256)		**OR**	**95%CI**	**p**	**OR**	**95%CI**	**p**	**OR**	**95%CI**	**p**	**OR**	**95%CI**	**p**
Thinness	324	0.54	0.02 12.64	0.70	0.85	0.27 2.74	0.79	0.50	0.15 1.68	0.26	0.66	0.21 2.04	0.47
Overweight	496	3.51	0.04 333.8	0.59	2.04	0.25 16.75	0.51	2.57	0.32 20.45	0.37	3.22	0.42 24.90	0.26
Obese	201	0.04	<0.001 21.42	0.31	0.41	0.07 2.54	0.34	0.37	0.06 2.33	0.29	0.52	0.11 2.57	0.42
Overweight or obese	697	0.60	0.03 13.52	0.75	1.03	0.27 3.96	0.96	1.21	0.32 4.53	0.78	1.52	0.42 5.45	0.52

* Posterior probabilities for TTT homozygotes were vey low (sum=29)

(cont.)

Recessive model

Phenotype at T0 – SNPs (presence vs absence)

Phenotype at T0	N	rs6827359 C (24.3%)				rs12500837 C (6.0%)				rs9999653 T (29.4%)			
	N	beta		se	p	beta		se	p	beta		se	p
Bmi (ln)	4528	-0.18	±	0.08	0.014	-0.16	±	0.14	0.215	-0.17	±	0.08	0.355
Bmi z-score	4528	-0.11	±	0.04	0.008	-0.08	±	0.08	0.319	-0.10	±	0.04	0.457
Weight, Kg (ln)	4528	-0.35	±	0.15	0.016	-0.29	±	0.28	0.501	-0.32	±	0.16	0.447
Fat mass, %	4463	-0.22	±	0.10	0.028	-0.20	±	0.18	0.274	-0.24	±	0.11	0.217
Fat-free mass, %	4454	-0.11	±	0.08	0.140	-0.07	±	0.14	0.628	-0.06	±	0.08	0.825
Tricipital+subscapular skinfolds, mm (ln)	4,391	-0.40	±	0.24	0.071	-0.71	±	0.44	0.063	-0.48	±	0.26	0.185
Tricipital skinfold, mm (ln)	4416	-0.20	±	0.13	0.095	-0.41	±	0.24	0.065	-0.21	±	0.14	0.186
Subscapular skinfold, mm (ln)	4413	-0.20	±	0.13	0.091	-0.27	±	0.23	0.124	-0.27	±	0.13	0.156
waist circumference, cm (ln)	4527	-0.28	±	0.20	0.135	-0.01	±	0.35	0.982	-0.35	±	0.20	0.669
hip circircumference, cm (ln)	4527	-0.44	±	0.19	0.017	-0.11	±	0.34	0.884	-0.40	±	0.20	0.128
waist-to-hip ratio	4526	0.002	±	0.002	0.334	0.001	±	0.003	0.774	0.000	±	0.002	0.137
arm circumference, cm (ln)	4497	-0.18	±	0.07	0.012	-0.17	±	0.13	0.194	-0.14	±	0.08	0.088
ref: normal weight (N=3234)		OR	95%CI		p	OR	95%CI		p	OR	95%CI		p
Thinness	478	1.11	0.89	1.38	0.37	0.90	0.59	1.38	0.64	1.12	0.90	1.38	0.87
Overweight	541	0.86	0.69	1.07	0.17	0.74	0.48	1.13	0.16	0.89	0.73	1.10	0.22
Obese	275	0.96	0.72	1.30	0.80	0.84	0.48	1.47	0.53	0.87	0.65	1.16	0.68
Overweight or obese	816	0.88	0.73	1.06	0.18	0.77	0.54	1.10	0.16	0.88	0.73	1.05	0.24

Phenotype at T0 – Most frequent haplotype (TTC , 43.9%) as reference

Phenotype at T0	CCC/CCC (0.02%)				CCT/CCT (5.0%)				CTT/CTT (6.2%)				TTT/TTT (0.7%)			
	beta		se	p	beta		se	p	beta		se	p	beta		se	p
Bmi (ln)	-0.41	±	2.19	0.92	-0.20	±	0.15	0.168	-0.08	±	0.14	0.49	1.13	±	0.41	0.009
Bmi z-score	0.07	±	1.23	0.96	-0.10	±	0.08	0.257	-0.08	±	0.08	0.32	0.57	±	0.23	0.012
Weight, Kg (ln)	-1.93	±	4.40	0.70	-0.42	±	0.30	0.311	-0.25	±	0.27	0.29	1.48	±	0.83	0.09
Fat mass, %	-1.22	±	2.87	0.67	-0.29	±	0.20	0.149	-0.19	±	0.18	0.30	1.36	±	0.54	0.012
Fat-free mass, %	-0.71	±	2.21	0.75	-0.10	±	0.15	0.51	-0.06	±	0.14	0.65	0.12	±	0.41	0.76
Tricipital+subscapular skinfolds, mm (ln)	-3.46	±	6.87	0.72	-0.81	±	0.48	0.084	0.06	±	0.43	0.83	3.17	±	1.33	0.012
Tricipital skinfold, mm (ln)	-1.95	±	3.73	0.68	-0.40	±	0.26	0.137	0.03	±	0.23	0.85	1.86	±	0.72	0.009
Subscapular skinfold, mm (ln)	-1.48	±	3.55	0.84	-0.37	±	0.25	0.097	0.02	±	0.22	0.73	1.92	±	0.68	0.007
waist circumference, cm (ln)	-1.46	±	5.56	0.86	-0.21	±	0.39	0.63	-0.18	±	0.35	0.63	2.14	±	1.05	0.045
hip circircumference, cm (ln)	0.33	±	5.37	0.84	-0.19	±	0.37	0.771	-0.41	±	0.34	0.21	2.01	±	1.01	0.06
waist-to-hip ratio	-0.032	±	0.048	0.51	-0.001	±	0.003	0.78	0.002	±	0.003	0.48	0.006	±	0.009	0.49
arm circumference, cm (ln)	-0.74	±	2.08	0.74	-0.17	±	0.14	0.228	-0.14	±	0.13	0.24	0.99	±	0.39	0.014
ref: normal weight (N=3234)	OR	95%CI		p	OR	95%CI		p	OR	95%CI		p	OR	95%CI		p
Thinness	-*	-	-	-	0.91	0.57	1.46	0.69	1.19	0.80	1.77	0.40	1.96	0.69	5.56	0.20
Overweight	-*	-	-	-	0.77	0.48	1.23	0.27	0.80	0.53	1.21	0.29	2.66	0.98	7.25	0.06
Obese	-*	-	-	-	0.76	0.40	1.41	0.38	1.09	0.63	1.86	0.76	3.45	0.91	13.16	0.07
Overweight or obese	-*	-	-	-	0.76	0.51	1.13	0.17	0.88	0.62	1.25	0.48	2.84	1.18	6.85	0.020

Phenotype at T0 – Haplotype carrying ancestral alleles (TTT, 7.2%) as reference

Phenotype at T0	CCC/CCC (0.02%)				CCT/CCT (5.0%)				CTT/CTT (6.2%)				TTC/TTC (19.5%)			
	beta		se	p	beta		se	p	beta		se	p	beta		se	p
Bmi (ln)	-0.40	±	2.20	0.92	-0.19	±	0.15	0.19	-0.07	±	0.14	0.54	0.08	±	0.08	0.37
Bmi z-score	0.07	±	1.23	0.95	-0.09	±	0.09	0.27	-0.07	±	0.08	0.34	0.03	±	0.05	0.56
Weight, Kg (ln)	-1.88	±	4.40	0.70	-0.38	±	0.31	0.36	-0.21	±	0.28	0.35	0.21	±	0.17	0.30
Fat mass, %	-1.18	±	2.87	0.68	-0.27	±	0.20	0.19	-0.16	±	0.18	0.37	0.15	±	0.11	0.19
Fat-free mass, %	-0.69	±	2.21	0.75	-0.09	±	0.15	0.57	-0.05	±	0.14	0.72	0.06	±	0.09	0.49
Tricipital+subscapular skinfolds, mm (ln)	-3.38	±	6.87	0.73	-0.76	±	0.48	0.11	0.11	±	0.44	0.72	0.36	±	0.27	0.15
Tricipital skinfold, mm (ln)	-1.90	±	3.74	0.69	-0.36	±	0.26	0.18	0.07	±	0.24	0.74	0.23	±	0.14	0.15
Subscapular skinfold, mm (ln)	-1.44	±	3.55	0.85	-0.34	±	0.25	0.13	0.05	±	0.23	0.62	0.18	±	0.14	0.11
waist circumference, cm (ln)	-1.42	±	5.57	0.86	-0.19	±	0.39	0.66	-0.16	±	0.35	0.66	0.16	±	0.21	0.52
hip circircumference, cm (ln)	0.43	±	5.37	0.82	-0.12	±	0.37	0.91	-0.34	±	0.34	0.30	0.39	±	0.21	0.07
waist-to-hip ratio	-0.033	±	0.048	0.50	-0.002	±	0.003	0.63	0.001	±	0.003	0.63	-0.003	±	0.002	0.12
arm circumference, cm (ln)	-0.70	±	2.08	0.75	-0.15	±	0.15	0.30	-0.12	±	0.13	0.34	0.14	±	0.08	0.07
ref: normal weight (N=3234)	OR	95%CI		p	OR	95%CI		p	OR	95%CI		p	OR	95%CI		p
Thinness	-*	-	-	-	0.73	0.37	1.41	0.35	1.51	0.78	2.92	0.22	1.05	0.69	1.59	0.81
Overweight	-*	-	-	-	0.68	0.35	1.32	0.26	0.87	0.46	1.65	0.66	1.05	0.69	1.58	0.83
Obese	-*	-	-	-	0.75	0.27	2.05	0.58	1.28	0.50	3.29	0.60	0.89	0.49	1.60	0.69
Overweight or obese	-*	-	-	-	0.68	0.38	1.21	0.19	0.96	0.55	1.68	0.89	0.99	0.70	1.42	0.97

Phenotype at T1 – SNPs (presence vs absence)

Phenotype at T1	N	rs6827359 C (24.4%)				rs12500837 C (5.8%)				rs9999653 T (29.7%)			
		beta		se	p	beta		se	p	beta		se	p
Bmi (ln)	3277	-0.23	±	0.11	0.036	-0.44	±	0.20	0.023	-0.16	±	0.11	0.332
Bmi z-score	3277	-0.10	±	0.05	0.052	-0.19	±	0.09	0.038	-0.05	±	0.05	0.458
Weight, Kg (ln)	3277	-0.54	±	0.26	0.033	-1.00	±	0.47	0.053	-0.37	±	0.27	0.458
Fat mass, %	3227	-0.42	±	0.18	0.017	-0.66	±	0.32	0.039	-0.32	±	0.18	0.082
Fat-free mass, %	3227	-0.12	±	0.12	0.314	-0.37	±	0.21	0.081	-0.07	±	0.12	0.831
Tric.+subsc. Skinfolds, mm (ln)	3180	-0.55	±	0.37	0.102	-1.10	±	0.67	0.053	-0.56	±	0.38	0.453
Tricipital skinfold, mm (ln)	3184	-0.37	±	0.19	0.058	-0.75	±	0.34	0.024	-0.30	±	0.19	0.545
Subscapular skinfold, mm (ln)	3183	-0.20	±	0.20	0.202	-0.36	±	0.36	0.131	-0.27	±	0.21	0.343
waist circumference, cm (ln)	3222	-0.46	±	0.28	0.101	-0.67	±	0.51	0.198	-0.34	±	0.29	0.938
hip circircumference, cm (ln)	3205	-0.49	±	0.26	0.074	-0.59	±	0.48	0.283	-0.35	±	0.27	0.461
waist-to-hip ratio	3200	0.001	±	0.002	0.765	-0.001	±	0.004	0.679	0.000	±	0.002	0.075
arm circumference, cm (ln)	3016	-0.25	±	0.11	0.018	-0.36	±	0.20	0.069	-0.15	±	0.11	0.136
ref: normal weight (N=2256)		OR	95%CI		p	OR	95%CI		p	OR	95%CI		p
Thinness	324	1.19	0.91	1.55	0.21	1.53	0.98	2.39	0.06	1.19	0.92	1.53	0.91
Overweight	496	0.83	0.65	1.05	0.13	0.67	0.41	1.08	0.10	0.87	0.70	1.09	0.53
Obese	201	0.84	0.58	1.20	0.34	0.97	0.51	1.86	0.94	0.81	0.57	1.15	0.19
Overweight or obese	697	0.83	0.67	1.03	0.09	0.77	0.51	1.16	0.21	0.86	0.70	1.05	0.24

Phenotype at T1 – Most frequent haplotype (TTC , 43.9%) as reference

Phenotype at T1	CCC/CCC (0.03%)				CCT/CCT (4.8%)				CTT/CTT (6.3%)				TTT/TTT (0.7%)			
	beta		se	p	beta		se	p	beta		se	p	beta		se	p
Bmi (ln)	-2.41	±	2.62	0.37	-0.37	±	0.22	0.09	-0.06	±	0.19	0.77	1.00	±	0.54	0.11
Bmi z-score	-0.99	±	1.22	0.42	-0.15	±	0.10	0.15	-0.04	±	0.09	0.69	0.30	±	0.25	0.24
Weight, Kg (ln)	-4.81	±	6.23	0.36	-0.87	±	0.51	0.16	-0.21	±	0.45	0.65	1.51	±	1.28	0.40
Fat mass, %	-3.09	±	4.21	0.46	-0.65	±	0.35	0.07	-0.10	±	0.31	0.74	1.59	±	0.87	0.07
Fat-free mass, %	-1.64	±	2.81	0.56	-0.26	±	0.23	0.27	-0.16	±	0.21	0.43	-0.10	±	0.58	0.87
Tric.+subsc. Skinfolds, mm (ln)	-7.95	±	8.76	0.39	-0.90	±	0.73	0.21	0.08	±	0.65	0.96	3.35	±	1.84	0.14
Tricipital skinfold, mm (ln)	-7.36	±	4.52	0.038	-0.58	±	0.37	0.16	-0.15	±	0.33	0.71	1.53	±	0.94	0.16
Subscapular skinfold, mm (ln)	-0.64	±	4.73	0.79	-0.34	±	0.40	0.28	0.22	±	0.35	0.55	1.81	±	1.00	0.19
waist circumference, cm (ln)	-3.95	±	6.69	0.59	-0.56	±	0.56	0.34	0.10	±	0.49	0.88	2.26	±	1.38	0.12
hip circircumference, cm (ln)	-5.13	±	6.33	0.41	-0.37	±	0.53	0.60	-0.28	±	0.46	0.55	1.54	±	1.30	0.30
waist-to-hip ratio	0.012	±	0.047	0.79	-0.002	±	0.004	0.55	0.006	±	0.003	0.08	0.013	±	0.010	0.18
arm circumference, cm (ln)	-2.06	±	2.54	0.39	-0.32	±	0.22	0.149	-0.26	±	0.19	0.15	0.93	±	0.55	0.12
ref: normal weight (N=2256)	OR	95%CI		p	OR	95%CI		p	OR	95%CI		p	OR	95%CI		p
Thinness	-*	-	-	-	1.44	0.88	2.36	0.15	1.46	0.91	2.33	0.12	1.52	0.49	4.74	0.47
Overweight	-*	-	-	-	0.71	0.42	1.22	0.21	1.02	0.67	1.57	0.91	0.31	0.04	2.40	0.26
Obese	-*	-	-	-	0.99	0.50	1.96	0.97	1.01	0.53	1.90	0.98	1.91	0.39	9.43	0.42
Overweight or obese	-*	-	-	-	0.81	0.51	1.27	0.35	1.02	0.69	1.49	0.94	0.66	0.18	2.36	0.52

Phenotype at T1 – Haplotype carrying ancestral alleles (TTT, 7.2%) as reference

Phenotype at T1	CCC/CCC (0.03%)				CCT/CCT (4.8%)				CTT/CTT (6.3%)				TTC/TTC (19.5%)			
	beta		se	p	beta		se	p	beta		se	p	beta		se	p
Bmi (ln)	-2.39	±	2.62	0.38	-0.35	±	0.22	0.11	-0.03	±	0.19	0.86	0.14	±	0.12	0.29
Bmi z-score	-0.98	±	1.22	0.42	-0.14	±	0.10	0.16	-0.03	±	0.09	0.73	0.03	±	0.05	0.54
Weight, Kg (ln)	-4.73	±	6.23	0.37	-0.79	±	0.52	0.20	-0.14	±	0.46	0.76	0.39	±	0.28	0.25
Fat mass, %	-3.01	±	4.21	0.47	-0.58	±	0.35	0.10	-0.03	±	0.31	0.93	0.39	±	0.19	0.042
Fat-free mass, %	-1.62	±	2.81	0.56	-0.24	±	0.24	0.32	-0.14	±	0.21	0.50	0.10	±	0.13	0.43
Tric.+subsc. Skinfolds, mm (ln)	-7.84	±	8.76	0.40	-0.80	±	0.74	0.25	0.19	±	0.65	0.86	0.61	±	0.40	0.24
Tricipital skinfold, mm (ln)	-7.30	±	4.52	0.039	-0.52	±	0.38	0.19	-0.09	±	0.33	0.79	0.30	±	0.20	0.31
Subscapular skinfold, mm (ln)	-0.57	±	4.73	0.78	-0.28	±	0.40	0.35	0.28	±	0.35	0.46	0.33	±	0.22	0.19
waist circumference, cm (ln)	-3.94	±	6.70	0.59	-0.55	±	0.56	0.34	0.11	±	0.50	0.89	0.13	±	0.30	0.84
hip circircumference, cm (ln)	-5.05	±	6.33	0.41	-0.29	±	0.53	0.69	-0.21	±	0.47	0.66	0.39	±	0.29	0.22
waist-to-hip ratio	0.011	±	0.047	0.81	-0.003	±	0.004	0.40	0.005	±	0.003	0.16	-0.004	±	0.002	0.06
arm circumference, cm (ln)	-2.02	±	2.54	0.40	-0.28	±	0.22	0.20	-0.22	±	0.19	0.22	0.20	±	0.12	0.12
ref: normal weight (N=2256)	OR	95%CI		p	OR	95%CI		p	OR	95%CI		p	OR	95%CI		p
Thinness	-*	-	-	-	1.09	0.53	2.24	0.81	2.07	0.93	4.61	0.08	1.22	0.74	2.04	0.44
Overweight	-*	-	-	-	0.69	0.32	1.49	0.34	1.52	0.74	3.11	0.25	0.95	0.61	1.49	0.82
Obese	-*	-	-	-	1.11	0.33	3.71	0.86	0.87	0.31	2.45	0.79	1.27	0.61	2.66	0.52
Overweight or obese	-*	-	-	-	0.83	0.42	1.63	0.58	1.27	0.69	2.36	0.45	1.02	0.68	1.53	0.91

* Posterior probabilties were very low
